# Supplementary material for: Colony morphogenesis regulates sporulation dynamics in bacterial biofilms
Source: bioRxiv. 2026 Feb 12:2026.02.11.705348. Preprint. [Version 1] doi: 10.64898/2026.02.11.705348 (PMC12919038; doi:10.64898/2026.02.11.705348)
Supplement: Supplement 5 [file NIHPP2026.02.11.705348v1-supplement-5.pdf]

Figure S1 Defective cell-cell signaling alters biofilm morphology

(A) Color photographs of the top surface of biofilms at 24 hpi. (B) Boxplots representing the change in biofilm radius from inoculation to 24 hpi. Central lines indicate medians, boxes enclose the middle 50% of the data, and whiskers indicate the minimum and maximum measurements; $n = 6$ biofilms. $^{***}p < 0.001$ by doubled-sided t-test on independent samples with unequal variance ($p = 4.24 \times 10^{-6}$ for 3610Δ*rapP*+*rapP*, $p = 3.14 \times 10^{-13}$ for PS-216+*rapP*).

Scale bars 3 mm.

Figure S2 Biofilm spreading is weakly dependent on EPS for strains without ***rapP*^{3610}**

(A-B) Color photographs of the top surface of biofilms at 24 hpi. (C) Boxplots representing the change in biofilm radius from inoculation to 24 hpi. Central lines indicate medians, boxes enclose the middle 50% of the data, and whiskers indicate the minimum and maximum measurements; $n = 12$ biofilms. $^{**}p < 0.01$ by doubled-sided t-test on independent samples with unequal variance ($p = 0.0049$ for 3610Δ*epsA-O*, $p = 0.0038$ for 3610Δ*rapP*Δ*epsA-O*, $p = 0.101$ for PS-216Δ*epsA-O*.

Scale bars 3 mm.

Figure S3 Tracking spread of sporulation activity through biomass

(A) Profiles of mScarlet-positive pixel count vs radial position (related to Figure 4A). One-dimensional gaussian smoothing ($\sigma = 50$) was used prior to peak detection. Vertical magenta lines indicate the detected peak of the smoothed data. Light magenta shading indicates the vegetative cell front, extending from the left base of the mScarlet peak to the leading edge of biomass. (B) Profiles of mean YFP/mScarlet vs radial position corresponding to the data shown in Figure 4C. Vertical green lines indicate the maximum value of smoothed profiles ($\sigma = 6$), excluding data outside the vegetative cell front.

Figure S4 Quantification of leading edge and spore-front velocities

(A) Tracking of biofilm leading edge and sporulation front positions. Magenta and green circles, respectively, indicate experimentally measured biomass leading edge and sporulation front positions. Central lines indicate interpolated values. Horizontal dotted lines mark the range of positions used for calculating velocity ratios. (B) Instantaneous velocity as a function of position for interpolated values in panel A. Gray shading corresponds to the range defined in A by dotted lines. (C) Calculated velocity ratios (sporulation front over expansion) plotted against the

Video S1. Propagating front of mScarlet-positive biomass (related to Figure 4)

Video running through timelapse frames of mScarlet radius-Z cross-section data. Data are from an independent biofilm compared to that in Figure 4. Images were straightened (ImageJ) to correct for substrate curvature.

Scale bar 100 x 50 µm.

Video S2. Propagating mScarlet and sporulation activity (related to Figure 4)

Video running through timelapse frames corresponding to Figures 4A and 4C. Magenta, mScarlet summed across z stacks; green, YFP/mScarlet for dual YFP- and mScarlet-positive pixels.

Scale bar 0.2 mm.

Video S3. Propagating mScarlet and sporulation activity for single-strain and co-culture biofilms (related to Figure 6)

Video running through timelapse frames of merged radius-Z cross-sections of mScarlet (magenta) and YFP/mScarlet (green) data for the representative biofilms shown in Figure 6. Images were straightened (ImageJ) to correct for substrate curvature.

Scale bar 500 x 100 µm.

Figure S1

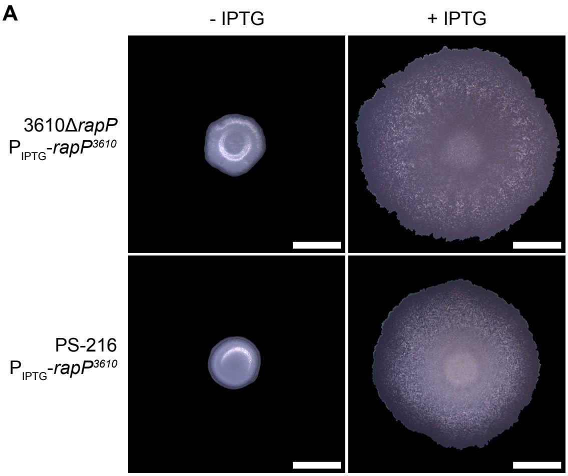
A
- IPTG
+ IPTG
3610Δ*rapP*
P_{IPTG}-*rapP*^{3610}
PS-216
P_{IPTG}-*rapP*^{3610}

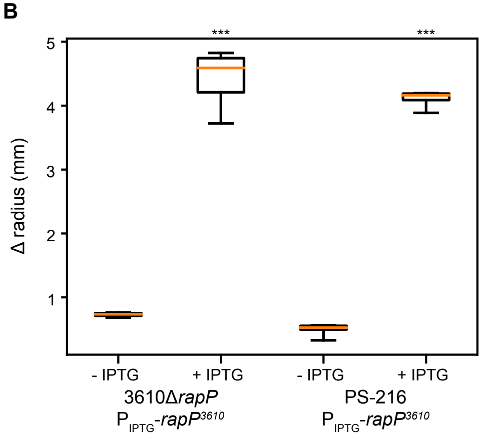
B
Δ radius (mm)
5
4
3
2
1
***
***
- IPTG
+ IPTG
- IPTG
+ IPTG
3610Δ*rapP*
P_{IPTG}-*rapP*^{3610}
PS-216
P_{IPTG}-*rapP*^{3610}

Figure S2

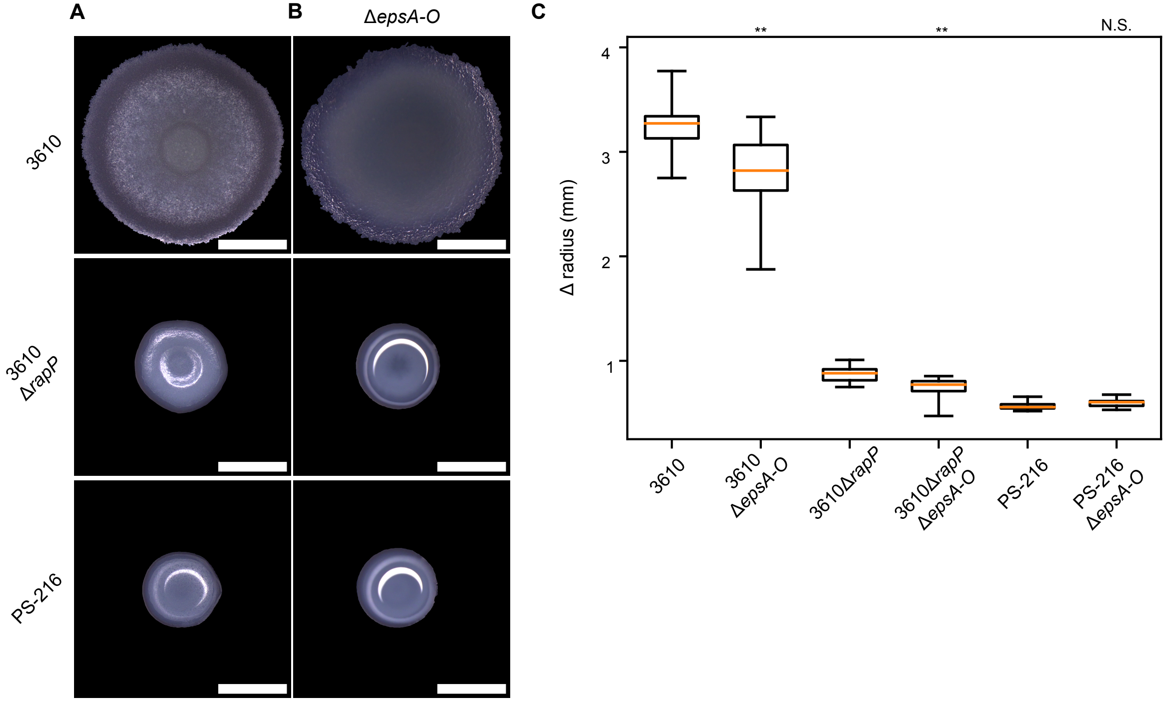
A
B
ΔepsA-O
C
3610
3610 ΔrapP
PS-216
**
**
N.S.
Δ radius (mm)
4
3
2
1
3610
3610 ΔepsA-O
3610ΔrapP
3610ΔrapP ΔepsA-O
PS-216
PS-216 ΔepsA-O

Figure S3

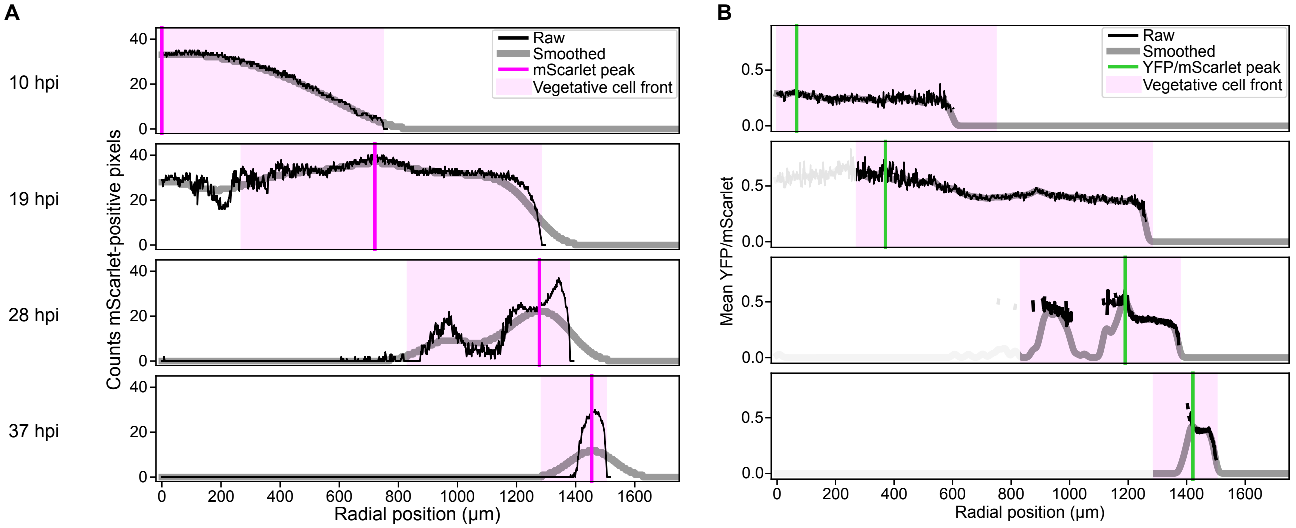
A
B
10 hpi
19 hpi
28 hpi
37 hpi
Counts mScarlet-positive pixels
Raw
Smoothed
mScarlet peak
Vegetative cell front
Radial position (μm)
Mean YFP/mScarlet
YFP/mScarlet peak
0
20
40
0.0
0.5
200
400
600
800
1000
1200
1400
1600

Figure S4

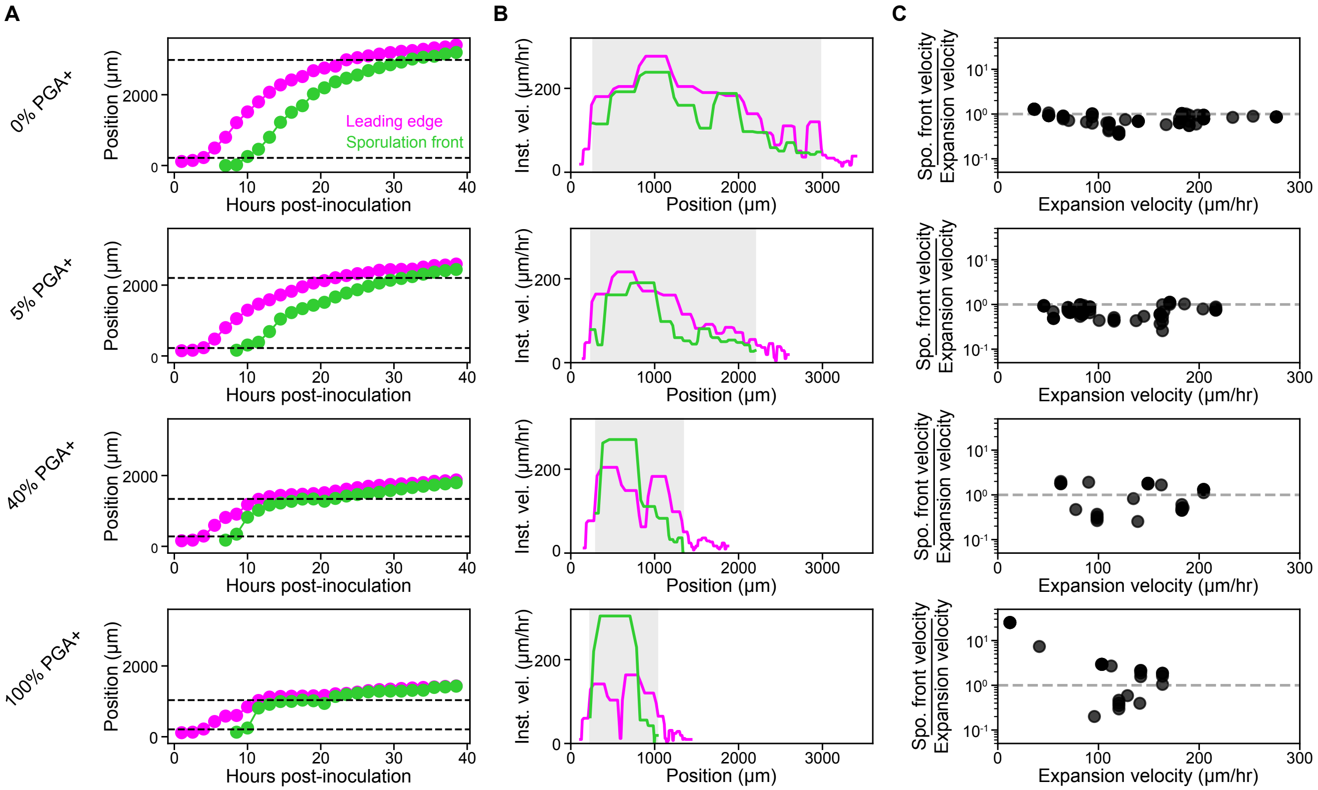
A
B
C
0% PGA+
5% PGA+
40% PGA+
100% PGA+
Position (µm)
Hours post-inoculation
Leading edge
Sporulation front
Inst. vel. (µm/hr)
Position (µm)
Spo. front velocity
Expansion velocity
Expansion velocity (µm/hr)
